# Yellow Fever in Pregnancy: A Comprehensive Review of the Clinical Implications and Vaccination in the Context of the 2024–2026 Americas Outbreak

**DOI:** 10.3390/tropicalmed11040092

**Published:** 2026-03-30

**Authors:** Alfonso J. Rodríguez-Morales, Katherine Acevedo-Jimenez, María Eugenia Guevara, Alicia Chang-Cojulun, José Brea-Del Castillo, Melissa Palmieri, Maria L. Avila-Agüero, Francisco Javier Membrillo de Novales, Carlos Torres-Martínez, Sandra X. Olaya, Sergio David Angulo, Jaime A. Cardona-Ospina, Roberto Debbag, Carlos Espinal, Maritza Cabrera, Jaime David Acosta-España, Darío S. López-Delgado, Marco A. Solarte-Portilla, Oscar Fraile, Tatiana Drummond, Rodrigo Nogueira Angerami, Flor M. Muñoz, Irene Benítez, Kleber Luz, María Alejandra López-Zambrano, Cristina Hernán-García, Daniel Leonardo Sánchez-Carmona, Lisette Cortes, Hernán Vargas, Lysien Zambrano, Danna Lucía Calderón-Medina, Diana Alejandra Hernández-Ramírez, Abraham Katime, Álvaro A. Faccini-Martínez, Leidy J. Medina-Lozano, Beatriz Elena Porras-Pedroza, Cristian Biscayart, Ana Carvajal, Lily M. Soto-Ávila, Marbelys Hernández, Rolando Ulloa-Gutierrez, Laura Naranjo-Lara, José Alejandro Mojica, Matthew H. Collins, Herberth Maldonado, Marco A. P. Safadi, Enrique Chacon-Cruz, José A. Suárez

**Affiliations:** 1 Faculty of Health Sciences, Universidad Cientifica del Sur, Lima 15067, Peru; 2 Grupo de Investigación Biomedicina, Faculty of Medicine, Fundación Universitaria Autónoma de las Américas-Institución Universitaria Visión de las Américas, Pereira 660003, Colombia; 3 Program of Family Medicine Specialization, Faculty of Medicine, Fundación Universitaria Autónoma de las Américas-Institución Universitaria Visión de las Américas, Pereira 660003, Colombia; 4 Faculty of Medicine, Universidad Centro-Occidental Lisandro Alvarado, Barquisimeto 3001, Venezuela; 5 Unidad Nacional de Oncologia Pediatrica (UNOP), Guatemala City 01013, Guatemala; 6 Pediatrics and Pediatric Infectious Diseases, Facultad de Ciencias de la Salud del Instituto Tecnológico de Santo Domingo, Santo Domingo 10602, Dominican Republic; 7 Municipal Health Department (SMS/SP), São Paulo Municipal Government, São Paulo 01509-020, Brazil; 8 Pediatric Infectious Diseases Department, Hospital Nacional de Niños, San José 111221, Costa Rica; avilaaguero@gmail.com; 9 Center for Infectious Disease Modeling and Analysis (CIDMA), Yale University, New Haven, CT 06520, USA; 10 CBRN and Infectious Diseases Department, Hospital Central de la Defensa Gómez Ulla, 28047 Madrid, Spain; 11 Department of Pediatrics, Universidad El Bosque, Cafettor Médica SAS, Bogotá 110311, Colombia; 12 Gynecology, Clinica Comfamiliar, Pereira 660001, Colombia; 13 Research and Teaching Unit, Salud Comfamiliar, Pereira 660001, Colombia; 14 Division of Infectious Diseases and Vaccinology, School of Public Health, University of California, Berkeley, CA 94720, USA; 15 Grupo de Investigación en Infecciones Emergentes y Medicina Tropical, Instituto para la Investigación en Ciencias Biomédicas, Sci-Help, Pereira 660003, Colombia; 16 Latin-American Vaccinology Society, Buenos Aires C1425AWK, Argentina; 17 Robert Stempel College of Public Health and Social Work, Florida International University, Miami, FL 33199, USA; 18 Centro de Investigación de Estudios Avanzados del Maule (CIEAM), Universidad Católica del Maule, Talca 3480094, Chile; 19 Health Sciences Faculty, Universidad Internacional SEK (UISEK), Quito 170120, Ecuador; 20 School of Medicine, Pontificia Universidad Católica del Ecuador, Quito 170525, Ecuador; 21 Institute of Microbiology, Friedrich Schiller University Jena, 07743 Jena, Germany; 22 Research Group of Emerging and Neglected Diseases, Ecoepidemiology and Biodiversity, Health Sciences Faculty, Universidad Internacional SEK (UISEK), Quito 170120, Ecuador; 23 Centro de Investigación para la Salud en América Latina (CISeAL), Pontificia Universidad Católica del Ecuador, Quito 170143, Ecuador; 24 Hospital Universitario Departamental de Nariño, Pasto 520006, Colombia; darkeio92@gmail.com (D.S.L.-D.);; 25 Hospital Nuestra Señora de Sonsoles (HNSS), 05071 Avila, Spain; 26 Service of Pediatric Infectious Diseases, Hospital Universitario de Caracas, Caracas 1053, Venezuela; 27 Núcleo de Vigilância Epidemiológica, Seção de Epidemiologia Hospitalar, Hospital de Clínicas, Universidade Estadual de Campinas, Campinas 13083-894, Brazil; rodrigo.angerami@gmail.com; 28 Division of Infectious Diseases, Department of Pediatrics, Baylor College of Medicine, Houston, TX 77030, USA; 29 Department of Molecular Virology and Microbiology, Baylor College of Medicine, Houston, TX 77030, USA; 30 Instituto de Previsión Social, Ministerio de Salud Pública y Bienestar Social, Asunción 1201, Paraguay; 31 Facultad de Ciencias de la Salud, Universidad Católica Nuestra Señora de Asunción, Asunción 1718, Paraguay; 32 Instituto de Medicina Tropical da Universidade Federal do Rio Grande do Norte, Natal 59078-970, Brazil; klebergluz@gmail.com; 33 Subdirección General de Prevención y Promoción de la Salud, Consejería de Sanidad, Autonomous Community of Madrid, C/López de Hoyos, 35, 28002 Madrid, Spain; 34 Department of Preventive Medicine and Public Health, University of Valladolid, 47005 Valladolid, Spain; 35 Preventive Medicine and Public Health Service, Hospital Clínico Universitario de Valladolid, 47003 Valladolid, Spain; 36 Unidad de Terapia Intensiva de Adultos, Centro Clínico Maternidad Leopoldo Aguerrevere, Caracas 1061, Venezuela; 37 Dirección de Salud Pública, Secretaria de Salud del Tolima, Ibague 730001, Colombia; 38 Grupo de Investigación Clínica y Medicina Traslacional, Hospital Federico Lleras Acosta, Ibague 730002, Colombia; 39 Grupo de Inmunologia Molecular, Universidad del Quindío, Armenia 630004, Colombia; 40 Department of Morphological Sciences, School of Medical Sciences, Universidad Nacional Autónoma de Honduras, Tegucigalpa 11101, Honduras; lysien.zambrano@unah.edu.hn; 41 Service of Internal Medicine-Research Line IMPACTA-Salud, Hospital Federico Lleras Acosta, Ibague 730001, Colombia; 42 Faculty of Medicine, Fundación Universitaria Navarra (UNINAVARRA), Neiva 410010, Colombia; 43 Programa de Medicina, Universidad del Magdalena, Santa Marta 470004, Colombia; 44 Servicio de Infectología, Hospital Universitario Julio Méndez Barreneche, Santa Marta 111071, Colombia; 45 Comité de Medicina Tropical, Zoonosis y Medicina del Viajero, Asociación Colombiana de Infectología (ACIN), Bogotá 110111, Colombia; 46 Servicio de Infectología, Hospital Militar Central, Bogotá 110231, Colombia; 47 Facultad de Medicina, Universidad Militar Nueva Granada, Bogotá 110111, Colombia; 48 Programa de Especialización en Infectología, Universidad Nacional de Colombia, Bogotá 111321, Colombia; 49 Servicio de Medicina Interna, Hospital Militar Central, Bogotá 110231, Colombia; 50 Servicio de Medicina Interna, Clínica del Country, Clínica la Colina, Hospital Universitario Nacional de Colombia, Universidad Nacional de Colombia, Bogotá 111321, Colombia; 51 Facultad de Medicina, Fundación Universitaria Sanitas, Bogotá 110911, Colombia; dramedtrop@gmail.com; 52 Fundación GESICA (Grupo de Estudios Sobre Investigación Clínica en Argentina), Buenos Aires C1121ABE, Argentina; 53 Posgrado de Infectología, Hospital Universitario de Caracas, Universidad Central de Venezuela, Caracas 1050, Venezuela; 54 Sección de Inmunología, Instituto de Medicina Tropical, Facultad de Medicina, Universidad Central de Venezuela, Caracas 1050, Venezuela; 55 Clínica de la Mujer, Del Sur Policlínica, El Tigre 6050, Venezuela; 56 Servicio de Aislamiento, Hospital Nacional de Niños “Dr. Carlos Sáenz Herrera”, Centro de Ciencias Médicas, Caja Costarricense de Seguro Social (CCSS), San José 111221, Costa Rica; rolandoug@gmail.com; 57 Instituto de Investigación en Ciencias Médicas UCIMED (IICIMED), San José 111221, Costa Rica; 58 Cátedra de Pediatría, Facultad de Medicina, Universidad de Ciencias Médicas (UCIMED), San José 111221, Costa Rica; 59 Sistema Nacional de Investigación-SENACYT, Panama City 0819-07289, Panama; 60 Infectotrópico, S. A., Panama City 0819-07289, Panama; 61 Ministry of Health, Bogota 111411, Colombia; 62 Division of Infectious Diseases, Department of Medicine, Emory University School of Medicine, Atlanta, GA 30322, USA; matthew.collins@emory.edu; 63 Centro de Estudios en Salud, Universidad del Valle de Guatemala, Guatemala City 01015, Guatemala; hgmaldonado@uvg.edu.gt; 64 Department of Paediatrics, Santa Casa de São Paulo School of Medical Sciences, São Paulo 01221-020, Brazil; masafadi@uol.com.br; 65 Think Vaccines LLC, Houston, TX 77005, USA; enrique.chacon@thinkvaccines.org

**Keywords:** yellow fever, pregnancy, maternal health, arboviral diseases, vaccination, safety, effectiveness

## Abstract

Yellow fever remains a major public health threat in endemic and re-emerging regions of Africa and South America, with recent outbreaks highlighting persistent gaps in prevention and surveillance. Pregnant women represent a particularly vulnerable population, yet the epidemiology, clinical impact, and preventive strategies for yellow fever in pregnancy are insufficiently characterized. Physiological and immunological changes during gestation may influence host responses to infection; however, current evidence does not demonstrate increased susceptibility to or severity of yellow fever during pregnancy. Adverse materno-fetal outcomes, including miscarriage, stillbirth, preterm birth, and, in rare cases, perinatal transmission, have been reported but remain poorly characterized. Diagnostic challenges, overlapping clinical presentations with other arboviral and hepatic diseases, and limited access to specialized care further complicate clinical management in many endemic settings. This perspective provides a comprehensive overview of yellow fever in pregnancy during the 2024–2026 outbreak in the Americas, including a risk-stratification framework for prevention. We summarize current evidence on epidemiology, pathophysiology, diagnosis, and supportive care, and examine prevention strategies with particular emphasis on vaccination. Accumulated observational evidence and substantial real-world experience have not demonstrated an increased risk of serious adverse events and generally support the effectiveness of yellow fever vaccination during pregnancy when administered with appropriate clinical judgment. In high-risk settings, the benefits of maternal immunization clearly outweigh theoretical concerns, supporting a flexible, risk-based approach, despite relatively limited evidence. We also discuss national and international policies, post-pregnancy booster recommendations, and the importance of integrating vaccination assessment into antenatal care. Finally, we highlight critical knowledge gaps and research priorities, including the need for prospective registries and strengthened pharmacovigilance. Coordinated clinical and public health strategies are essential to protect maternal and neonatal health and to reduce the burden of yellow fever in endemic and re-emerging settings.

## 1. Introduction

Yellow fever (YF) is one of the most severe mosquito-borne viral diseases worldwide, causing recurrent outbreaks with substantial morbidity and mortality in endemic regions of Africa and South America [1]. Despite the availability of an effective live-attenuated vaccine, recent years have witnessed a marked resurgence of YF in several countries [2], particularly in South America [3]. Expanding sylvatic transmission [4], ecological disruption [5,6], population mobility [7], and suboptimal vaccination coverage have contributed to renewed epidemic activity [8]. These dynamics have increased the risk of both rural and peri-urban transmission, raising concerns about the potential reemergence of the urban YF cycle and international spread [9]. In South America, 443 cases and 193 deaths (~44%) have been confirmed during the 2024–2026 outbreak up to 14 March 2026, with cases in 7 countries (Table 1) [7,10,11]. Currently, PAHO/WHO surveillance platforms do not routinely report pregnancy-specific case data (this would also be important in other conditions, such as measles and dengue).

During the 2024–2026 South American outbreak, the highest incidence was observed in Guyana (0.48 cases per 100,000 population), whereas the highest case-fatality rate was observed in Ecuador (72.7%), although this was based on a small number of reported cases. These differences likely reflect heterogeneity in outbreak size, surveillance sensitivity, healthcare access, and delays in diagnosis and referral across affected countries. The incidence rate in Guyana has been 8 times higher than in Brazil and Ecuador, despite the low denominators; however, in Colombia, with higher denominators, it has been 5 times higher than in Brazil (Table 1).

Pregnant women represent a particularly vulnerable population in the context of YF infection (Table 2) [7,12,13,14,15,16,17]. However, it remains uncertain whether pregnancy modifies the clinical spectrum or severity of YF in affected women compared with non-pregnant individuals. Regarding fetal risk, available evidence remains extremely limited. Current reviews indicate that only two cases of confirmed vertical transmission have been reported, both characterized by initially asymptomatic neonates who subsequently developed severe disease with fever, multiorgan failure, and coagulopathy, ultimately resulting in death [16].

Physiological and immunological changes during pregnancy may influence disease severity, clinical presentation, and immune responses, potentially increasing the risk of adverse materno-fetal outcomes [12,15]. Available evidence, although limited, suggests that YF during pregnancy may be associated with severe maternal illness, miscarriage, stillbirth, preterm delivery, and, in rare cases, vertical transmission [7,12,13,14,15,16,17]. However, the true burden of YF in pregnancy remains poorly characterized, largely due to underreporting, limited surveillance, and the scarcity of prospective studies [18].

On the other hand, clinical management and prevention of YF in pregnant women pose significant challenges. Diagnostic difficulties, overlapping clinical features with other arboviral infections, and restricted therapeutic options complicate timely recognition and appropriate care [19].

Moreover, the use of live-attenuated YF vaccines during pregnancy remains a controversial critical issue. It requires careful risk–benefit assessment, particularly in outbreak settings or when travel to endemic areas is unavoidable [20,21]. There is data on this matter, as documented in a 2000 study that reported inadvertent vaccination of 480 pregnant women in the early stages of pregnancy as part of a vaccination campaign against YF, with no safety concerns; even applied during the first trimester, no malformations, complications to the central nervous system, nor adverse perinatal results were observed [22].

In the context of climate change, increased human mobility, and persistent gaps in immunization programs, the intersection between YF and maternal health has become an increasingly important public health concern [23]. This article provides a comprehensive perspective on YF in pregnancy. It addresses epidemiology, clinical impact, diagnostic and management considerations, and prevention strategies, with particular emphasis on vaccination policies and practical guidance. By synthesizing current evidence and identifying key knowledge gaps, we aim to inform clinicians, researchers, and policymakers and support the development of more effective maternal health and disease prevention strategies in YF-endemic and re-emerging settings. We searched studies and articles across multiple databases, including Web of Science, Scopus, PubMed, SciELO, LILACS, Latindex, and ScienceDirect. We were supported by an evidence assessment using the OpenEvidence and VeraHealth platforms; however, this is not a systematic or scoping review. We used a search strategy designed to ensure both breadth and specificity, in line with the objectives of this review. Terms including “Yellow fever (YF)”, “pregnancy and YF”, “Epidemiology of YF”, “Pathophysiology and Pregnancy-Specific Issues”, “diagnosis of YF in pregnant”, “Prevention Strategies in Pregnancy”, “YF Vaccination in Before and During Pregnancy”. Eligible studies were those presenting original data; articles were excluded if they contained incomplete or overlapping datasets, lacked access to the full text. These criteria were implemented to maintain methodological rigor and ensure the validity of the synthesized evidence.

**Table 2:** Reported Materno-fetal outcomes associated with yellow fever infection during pregnancy [13,17,24,25,26].

Reference (Study Type)	Country	Years	Study Design	Gestational Age at Infection	Maternal Outcomes	Fetal/Neonatal Outcomes	Key Findings
Historical clinical/outbreak descriptions, including pregnancy [24]	South America and Africa	20th century (reviewed)	Narrative historical/clinical review	Not consistently reported	Severe hepatitis, hemorrhage, shock, and death in severe cases	Pregnancy loss reported in historical literature	Contextualizes early observations and biologic plausibility
Perinatal transmission of wild-type yellow fever [13]	Brazil	2009 (published 2011)	Case report/correspondence	Peripartum	Severe maternal illness	Neonatal infection with fulminant YF and death	Key documented perinatal transmission event
Perinatal yellow fever with maternal and neonatal fatality [17]	Brazil	2019	Case report	Peripartum	Fulminant hepatitis and maternal death	Neonatal liver failure and death; PCR positive	Laboratory-confirmed severe perinatal transmission
Summary of perinatal transmission events [26]	Global	Current guidance	Authoritative guidance	Peripartum	Maternal symptomatic YF near delivery	Two documented infant infections with fatal outcomes	Supports the rarity but severity of perinatal transmission
Synthesis of yellow fever epidemiology and transmission [25]	Global	2020	Review/synthesis	N/A	Summarizes severe disease spectrum	Notes vertical/perinatal transmission in context	Supporting synthesis (not primary cohort)

## 2. Epidemiology of YF in Pregnancy

YF remains endemic in large areas of sub-Saharan Africa and tropical South America, where millions of women of reproductive age live in settings with ongoing or periodic viral transmission [27]. Although vaccination has substantially reduced disease burden in many countries, recurrent outbreaks continue to occur, driven by sylvatic transmission cycles, ecological changes, population mobility, and gaps in immunization coverage [28]. In this context, pregnant women residing in or traveling to endemic regions remain at risk of exposure, particularly in rural, peri-urban, and forest-fringe environments where vector density is high [29].

Reliable epidemiologic data on the incidence and outcomes of YF in pregnancy remain scarce (Table 2) [7,12,13,14,15,16,17]. Most national surveillance systems do not routinely disaggregate cases by pregnancy status, and mild or asymptomatic infections are likely underreported. The absence of routinely reported age-, sex-, and pregnancy-specific surveillance data also limits the ability to generate reliable modeled estimates of the burden of yellow fever in pregnant women during recent outbreaks. Consequently, available epidemiological data are largely derived from outbreak investigations, hospital-based case series, and sporadic case reports. These sources suggest that infection during pregnancy is uncommon in absolute numbers but likely underestimated, especially in regions with limited access to diagnostic testing and prenatal care [30,31]. Nevertheless, in Colombia during the current 2024–2026 outbreak, as of February 3, 2026, 19.5% of cases have been confirmed among women (https://bit.ly/4a0Jjxi) (accessed on 9 February 2026). A similar situation has been observed in Peru, with 16.2% of the 2024–2026 confirmed cases among women (https://www.dge.gob.pe/sala-fiebre-amarilla/tablero.html) (accessed on 9 February 2026). In Brazil, 10.2% of the 2024–2026 confirmed cases occurred in women (https://www.gov.br/saude/pt-br/composicao/svsa/cnie/painel-febre-amarela) (accessed on 9 February 2026). However, among the total confirmed cases reported in Colombia between 2007 and 2023, only 1 case (4%) was in a female (https://www.sispro.gov.co/Pages/Home.aspx) (accessed on 9 February 2026). Although countries may record pregnancy status, it is not displayed publicly in surveillance systems. These proportions indicate that a notable share of infections occurred among women of reproductive age, although pregnancy status is not reported, and no inference about pregnancy-specific risk can be made. At present, reliable estimation of the burden of YF in pregnancy remains difficult because most available surveillance systems and outbreak reports do not publicly show the pregnancy status, gestational age, or age-specific denominators for women of reproductive age, even though in countries (e.g., Brazil or Colombia) the pregnancy status is a mandatory piece of information for reporting any suspected case. In this sense, assuming that every confirmed case has the gestational status documented in the notification and that there is no data on cases among pregnant women, it can be inferred that there were no cases of yellow fever among pregnant women in 2025–2026. As a result, indirect modeling based on general pregnancy rates in affected populations would require strong assumptions and could generate misleading estimates.

Historical and contemporary outbreaks in South America and Africa have intermittently documented cases among pregnant women, often in the context of large epidemics affecting predominantly unvaccinated populations [32]. In these settings, pregnant women are typically exposed through occupational activities, agricultural work, proximity to forested areas in the household, or travel to endemic zones [33,34]. Urbanization and the expansion of peri-urban settlements have further increased contact between human populations and sylvatic transmission cycles, raising concerns about broader exposure among women of childbearing age [35].

Sociodemographic and structural determinants also influence exposure risk and access to prevention in endemic areas [7]. Lower vaccination coverage, geographic isolation, low adherence to and attention from healthcare professionals regarding the recommendation for vaccination of women of childbearing age before pregnancy, and, in outbreak scenarios, the possibility of vaccination even during pregnancy, and barriers to healthcare access may increase vulnerability to infection among women of reproductive age, particularly in rural and forest-adjacent communities where sylvatic transmission occurs [36,37].

Travel-related exposure represents an additional epidemiological dimension [1,7,19,20]. Pregnant travelers from non-endemic countries may be exposed during visits to endemic areas for tourism, work, humanitarian missions, or family-related travel [1,7,19,20]. Although the absolute number of cases in this group remains low, the risk is amplified in outbreak settings or during prolonged stays in high-transmission zones. Increasing international mobility has therefore expanded the geographic scope of concern beyond traditionally endemic countries [1,7,19,20].

Temporal trends in YF epidemiology also have implications for pregnancy-related risk [26,38]. Recent decades have seen a resurgence of outbreaks associated with deforestation, climate variability, and changes in vector ecology [5,6]. These factors have facilitated the spread of competent mosquito vectors and prolonged transmission seasons, potentially increasing cumulative exposure among pregnant women [39]. Moreover, fluctuating vaccine supply, logistical challenges, and vaccine hesitancy have contributed to persistent immunity gaps in some regions [40].

Overall, the epidemiology of YF in pregnancy is characterized by limited data, potential underrecognition, and marked geographic and social heterogeneity [12,14,16,18,19,20,21]. While reported cases remain relatively infrequent [17], the true burden is likely higher than currently appreciated. Strengthening surveillance systems, integrating pregnancy status into routine reporting, and improving access to preventive services are essential to better define and address the risk of YF among pregnant populations in endemic and re-emerging settings [12,14,16,18,19,20,21]. The absence of publicly displayed, routinely reported age-, sex-, and pregnancy-specific surveillance data also limits the ability to generate reliable modeled estimates of the burden of yellow fever in pregnant women during recent outbreaks, even though it is collected in surveillance in some countries.

Understanding the epidemiological context of yellow fever in pregnancy provides an important foundation for examining the physiological and immunological mechanisms that may influence host responses to infection during gestation.

## 3. Pathophysiology and Pregnancy-Specific Issues

**Figure 1:**
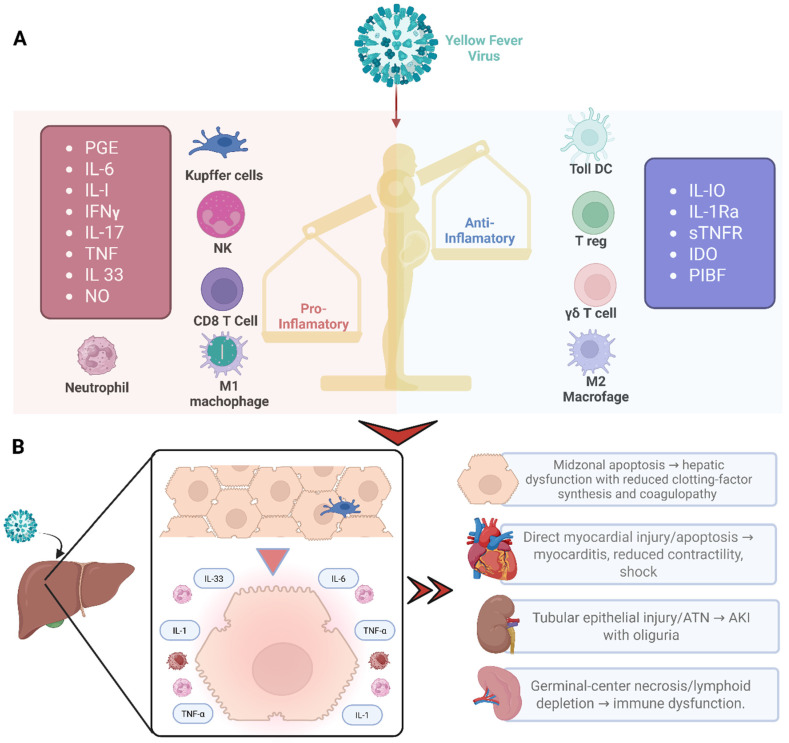
Immune balance during the tolerant phase of pregnancy and proposed multiorgan pathophysiology of severe yellow fever. (**A**) Conceptual and hypothetical model of the tolerant immune phase in pregnancy, highlighting regulatory cells and mediators on the anti-inflammatory side (tolerogenic DC, Treg, γδ T cells, M2 macrophages; IL-10, IL-1Ra, sTNFR, IDO, PIBF) versus pro-inflammatory effector cells and mediators (Kupffer cells, NK cells, CD8 T cells, M1 macrophages, neutrophils; PGE, IL-6, IL-1, IFN-γ, IL-17, TNF, IL-33, NO). The yellow fever virus is depicted as a disruptor that can shift this equilibrium toward inflammatory dominance. (**B**) Proposed sequence of severe yellow fever injury: intrahepatic infection and cytokine signaling (e.g., IL-1, IL-6, TNF-α, IL-33) contributing to hepatocellular dysfunction and systemic inflammation, followed by characteristic organ involvement—liver: midzonal hepatocyte apoptosis → hepatic dysfunction with reduced clotting-factor synthesis and coagulopathy; heart: direct myocardial injury/apoptosis → myocarditis, reduced contractility, shock; kidney: tubular epithelial injury/acute tubular necrosis (ATN) → acute kidney injury (AKI) with oliguria; spleen/lymphoid tissue: germinal-center necrosis/lymphoid depletion → immune dysfunction. Abbreviations: AKI, acute kidney injury; ATN, acute tubular necrosis; DC, dendritic cell; IDO, indoleamine 2,3-dioxygenase; IFN-γ, interferon gamma; IL-1Ra, interleukin-1 receptor antagonist; NK, natural killer; NO, nitric oxide; PGE, prostaglandin E; PIBF, progesterone-induced blocking factor; sTNFR, soluble TNF receptor; TNF-α, tumor necrosis factor alpha; Treg, regulatory T cell. This conceptual model illustrates proposed mechanisms through which pregnancy-related immune modulation and systemic inflammatory responses induced by the YF virus may interact to influence host responses to infection during pregnancy. Created with the help of BioRender (https://BioRender.com).

Pregnancy is characterized by complex physiological and immunological adaptations that may influence susceptibility to viral infections and disease severity (Figure 1). These include modulation of innate and adaptive immune responses, altered cytokine signaling, and a shift toward immune tolerance, which is necessary to maintain fetal viability [12,14,15]. While these mechanisms are essential for successful gestation, they may influence antiviral immune responses; however, current epidemiological data do not demonstrate that pregnancy increases susceptibility to or severity of yellow fever infection [12,14,15].

YF virus primarily targets hepatocytes, endothelial cells, and immune cells, leading to widespread hepatic injury, vascular dysfunction, and systemic inflammatory responses (Figure 1) [24]. In pregnant women, these effects may be amplified by pregnancy-related changes in hepatic metabolism, coagulation pathways, and cardiovascular physiology [24,25,41,42]. The combination of viral-induced liver failure, thrombocytopenia, and coagulopathy increases the risk of hemorrhagic complications, obstetric bleeding, and multi-organ dysfunction, posing significant threats to maternal survival [43,44].

The placenta serves as a critical interface between the maternal and fetal compartments and may function as both a barrier and a potential viral target. Although vertical transmission of YF appears to be rare, placental inflammation, endothelial injury, and altered perfusion may contribute to fetal hypoxia, growth restriction, and pregnancy loss. Experimental studies of other flaviviruses have shown that placental infection may occur through viral entry into trophoblasts and placental macrophages (Hofbauer cells), potentially mediated by surface receptors involved in flavivirus entry such as DC-SIGN and TAM family receptors, leading to local inflammation and disruption of the maternal–fetal interface [45,46,47]. In addition, systemic maternal illness, fever, hemodynamic changes, and metabolic disturbances can indirectly compromise fetal well-being [48,49,50].

The pregnancy-associated shift toward a T helper 2–dominant immune profile, which is essential for fetal tolerance, may theoretically attenuate virus-specific cytotoxic T-cell responses required for efficient clearance of flavivirus infections. This immunological modulation may contribute to suboptimal cellular immunity and accelerated waning of protective antibody titers following natural infection or vaccination. Consequently, some pregnant women may initially seroconvert but experience more rapid declines in antibody levels over time. This mechanism provides a plausible explanation for the reduced durability of vaccine-induced immunity observed in some studies of vaccination during pregnancy, although its relevance to susceptibility to wild-type yellow fever virus infection remains uncertain [51,52,53,54].

These pregnancy-specific physiological and immunological changes provide biological plausibility for altered host responses; however, current epidemiologic evidence does not demonstrate increased susceptibility or severity of yellow fever infection during pregnancy [14]. Importantly, available epidemiologic studies have not demonstrated higher case-fatality rates or clearly increased disease severity among pregnant women with yellow fever [14,51,52,53,54]. However, the absence of comparative cohort studies between pregnant and non-pregnant women limits the ability to definitively assess whether pregnancy modifies the clinical course of yellow fever.

These immunological adaptations may influence host responses to viral infections, potentially altering antiviral immunity during pregnancy. Similar mechanisms have been described in other arboviral infections during pregnancy. For example, dengue virus infection has been associated with exaggerated inflammatory responses and endothelial dysfunction that may increase the risk of severe disease. In contrast, the Zika virus is well recognized for its ability to infect placental trophoblasts and disrupt placental integrity. Although the mechanisms of yellow fever virus interaction with the placenta remain poorly characterized, experimental and clinical observations from related flaviviruses suggest that placental infection may occur through viral replication in trophoblastic cells, endothelial injury, and inflammatory cytokine signaling at the maternal–fetal interface. These processes could theoretically contribute to placental dysfunction, fetal hypoxia, and the rare cases of perinatal transmission reported in the literature [14,16,44,50,55,56,57,58]. However, direct mechanistic studies of YF virus infection in human placental tissue are currently lacking, and most proposed mechanisms are extrapolated from experimental or clinical observations in other flaviviral infections.

Importantly, no dedicated mechanistic studies have yet examined YF virus infection in human placental tissue.

## 4. Diagnosis in Pregnant Women

The diagnosis of YF in pregnant women is often challenging due to nonspecific early clinical manifestations and frequent overlap with other infectious and non-infectious conditions. Initial symptoms, including fever, headache, myalgia, nausea, and malaise, are indistinguishable from those of other arboviral infections, viral hepatitis (including hepatitis E and its well-known association with poor materno-fetal outcomes), malaria, leptospirosis, and obstetric complications such as preeclampsia with hepatic involvement, HELLP syndrome (Hemolysis, Elevated Liver enzymes, and Low Platelet count), and acute fatty liver of pregnancy (AFLP) (Table 3) [57,59,60,61]. For this reason, differential diagnostic frameworks, such as that summarized in Table 3, are useful for guiding early suspicion of diagnosis, clinical assessment, and selecting appropriate laboratory tests in endemic settings. However, definitive diagnosis ultimately relies on laboratory confirmation using molecular or serological methods. As a result, early high clinical suspicion is essential, particularly in women with recent exposure to endemic areas or ongoing outbreaks (e.g., Tolima in Colombia, 2024–2026) [58].

Laboratory confirmation relies primarily on molecular and serological methods [58]. In clinical practice, during the viremic phase, reverse transcription polymerase chain reaction allows direct detection of viral RNA and provides high diagnostic specificity. After the acute phase, serological testing for virus-specific antibodies becomes the main diagnostic tool. However, serological interpretation, even for IgM antibodies, may be complicated by cross-reactivity with other flaviviruses, prior vaccination, and pre-existing immunity, which are common in endemic regions [65].

In areas with recent vaccination campaigns, detection of YF–specific IgM may reflect recent immunization rather than acute infection, and confirmatory testing with plaque-reduction neutralization assays may be required in selected cases [66].

Pregnancy-related physiological changes, including hemodilution, altered immune responses, and modifications in liver enzyme levels, may further obscure diagnostic interpretation. In addition, limited access to advanced laboratory facilities in resource-constrained settings often delays confirmation. Consequently, clinical, epidemiological, and laboratory information must be integrated to support timely diagnosis. Early identification and accurate diagnosis of YF are critical to guide appropriate supportive management, fetal monitoring, and public health interventions aimed at reducing transmission and preventing adverse maternal and neonatal outcomes [67,68].

Once YF is suspected or confirmed, timely clinical management, monitoring, and intensive supportive care are essential to reduce maternal complications and optimize fetal and neonatal outcomes.

## 5. Clinical Management and Supportive Care

There is no specific antiviral therapy for YF, and clinical management in pregnant women is primarily based on early recognition and comprehensive supportive care. Given the potential for rapid clinical deterioration, suspected or confirmed cases should be managed in healthcare facilities with capacity for close clinical and obstetric monitoring and advanced supportive interventions. Initial management focuses on stabilizing vital signs, maintaining adequate hydration, correcting electrolyte imbalances, and maintaining hemodynamic stability [65,69].

Maternal hyperthermia itself may increase uterine contractility and has been associated with adverse obstetric and neurodevelopmental outcomes [65,70]. Substantial evidence suggests that fever during pregnancy, irrespective of the underlying pathogen, may represent a risk factor for neurodevelopmental disorders, autism spectrum disorders, and congenital cardiovascular malformations [65,70]. Nevertheless, results remain heterogeneous, and not all studies have demonstrated a significant increase in risk. These findings highlight the importance of investigating the causes of fever and implementing timely antipyretic and supportive measures in pregnant women with suspected or confirmed YF [65,70].

Acute liver failure, renal impairment, metabolic disturbances, and coagulopathy frequently complicate severe disease [71]. Regular monitoring of liver enzymes, renal function, coagulation parameters, and platelet counts is essential for early detection of organ dysfunction [72]. Blood products, vitamin K, and careful transfusion support may be required in the presence of significant bleeding or severe thrombocytopenia. Nephrotoxic and hepatotoxic medications should be avoided whenever possible [65,73].

In selected critically ill patients with refractory disease, adjunctive therapies such as intravenous immunoglobulin and intensive therapeutic plasma exchange have been proposed as rescue interventions in addition to standard supportive care, with emerging evidence suggesting potential benefit in severe forms of YF [65,74].

Obstetric management should be individualized and coordinated within a multidisciplinary team involving infectious disease specialists, obstetricians, intensivists, and neonatologists. Continuous fetal monitoring is recommended in viable pregnancies, particularly in critically ill patients. Decisions regarding timing and mode of delivery must balance maternal clinical status, gestational age, and fetal well-being [12,14,16,18,19,20,21,49,68].

In cases of maternal instability, priority should be given to optimizing maternal condition, as this remains the most effective strategy for improving fetal outcomes [12,14,16,18,19,20,21,49,68]. Pregnant women in endemic areas with active yellow fever transmission who present with fever, jaundice, and warning signs should be urgently referred to healthcare facilities with advanced supportive care capabilities, including dialysis and transplant services when available. Early recognition and timely transfer to higher-level care may be critical for improving outcomes in cases of fulminant hepatic failure [75], especially now, in countries with ongoing outbreaks in South America (2024–2026).

Postpartum follow-up is important for monitoring maternal recovery, assessing neonatal health, and providing appropriate counseling on future pregnancies and preventive measures [12,14,16,18,19,20,21,49,68].

In addition to maternal supportive care, careful consideration of fetal and neonatal risks is essential in the management of YF during pregnancy. Most documented cases described in the literature represent perinatal transmission occurring around the time of delivery rather than confirmed transplacental infection earlier in gestation. YF during pregnancy represents a significant clinical challenge because of the risk of vertical and perinatal transmission and the potential for severe neonatal complications. Although available evidence remains limited and is largely derived from isolated case reports, maternal–fetal transmission has been documented, particularly when maternal infection occurs late in pregnancy or around delivery. In such cases, neonatal infection may progress to fulminant disease. Published data describe a wide spectrum of outcomes ranging from asymptomatic congenital infection to fulminant neonatal disease and death. The best-documented perinatal case, reported in Brazil in 2009, described probable vertical transmission leading to neonatal hemorrhagic hepatitis, multi-organ failure, disseminated intravascular coagulation, seizures, and death within the first two weeks of life, underscoring the potential severity of congenital infection [13,76]. Despite these reported cases, the true incidence of vertical or perinatal transmission of the YF virus remains unknown. No large prospective cohorts or population-based surveillance studies have systematically evaluated maternal–fetal transmission, and available evidence is limited to isolated case reports and small clinical descriptions. Consequently, important questions remain unresolved, including the influence of gestational age on transmission risk, the spectrum of fetal outcomes following maternal infection, and the long-term prognosis of exposed infants. Establishing prospective pregnancy registries and integrating pregnancy status into yellow fever surveillance systems will be essential to address these knowledge gaps. Because the total number of pregnancies among infected women during outbreaks is unknown, the incidence of vertical or perinatal transmission cannot currently be estimated. Reliable risk estimates would require systematic recording of pregnancy status and prospective follow-up of maternal infections during outbreaks. Experimental animal studies have also demonstrated fetal growth restriction and reduced viability following early gestational exposure, suggesting increased vulnerability during the first trimester [77].

Cohort studies of inadvertent exposure to the 17D vaccine during pregnancy have not shown a consistent increase in major congenital malformations, although minor dysmorphisms have occasionally been reported, and rare cases of congenital infection following maternal vaccination have been documented [17,78,79,80]. In addition, the possibility of peripartum and lactational transmission, including through breast milk, further complicates risk assessment and clinical management [13,49,81].

Despite these concerns, there are currently no standardized fetal surveillance protocols specifically designed for pregnant women with confirmed or suspected YF. Existing guidelines do not define optimal monitoring intervals, diagnostic modalities, or evidence-based intervention thresholds [82]. Consequently, clinical management relies on general obstetric principles, including confirmation of maternal infection, close assessment of maternal stability, and individualized fetal surveillance using serial ultrasound for growth assessment, fetal wellbeing testing, and Doppler studies when clinically indicated [82]. In endemic settings, prevention remains central and focuses on preconception vaccination, individualized risk–benefit assessment during pregnancy, intensified surveillance of febrile pregnant women, and appropriate breastfeeding guidance following vaccination [49]. Prospective studies and pregnancy registries are urgently needed to determine true vertical transmission rates, characterize prenatal imaging findings, and establish evidence-based fetal and neonatal follow-up strategies to improve perinatal outcomes [13,76].

## 6. Prevention Strategies in Pregnancy

Prevention of YF and other arboviral diseases in pregnant women relies on an integrated approach combining vector control, personal protective measures, risk assessment, and targeted public health interventions [83], including attention to the immune status of pregnant women and people of childbearing potential. In endemic and high-risk areas, reducing exposure to infected mosquitoes remains a fundamental strategy [83,84]. Environmental management to eliminate breeding sites, regular insecticide application, and community-based vector control programs play a central role in limiting transmission [85]. At the individual level, pregnant women should be encouraged to use repellents approved for use during pregnancy, wear protective clothing, install window screens, and use bed nets, particularly in areas with intense vector activity [1,4,7,36]. At the individual level, women at risk of transmission may be advised to avoid pregnancy during periods of high transmission and outbreaks.

Risk reduction is especially important for women living in rural, peri-urban, and forest-adjacent settings, as well as those engaged in agricultural or outdoor occupations. Travel to endemic regions during pregnancy should be carefully evaluated, and non-essential travel should be postponed when feasible, particularly during outbreaks [86]. When travel is unavoidable, personalized counseling is essential to minimize exposure and ensure access to appropriate preventive measures [87].

Surveillance systems and early warning mechanisms, based on epizootic monitoring, climatic indicators, and human case detection, contribute to timely outbreak responses and targeted prevention efforts. Integrating YF prevention into routine antenatal care provides an opportunity to identify at-risk women, reinforce protective behaviors, and facilitate access to vaccination when appropriate. Strengthening community engagement, health education, and intersectoral collaboration is essential to sustain preventive strategies and reduce the burden of YF among pregnant populations [88,89]. In low-resource settings, maintaining adequate vaccine storage conditions and cold chain integrity represents an additional critical challenge, as failures in temperature control may compromise vaccine potency and undermine prevention efforts, particularly in remote and underserved communities [90,91,92,93].

Community perceptions of febrile illness and its management, as well as local patterns of human–animal interactions, play a central role in shaping the risk of YF transmission among pregnant women. Underestimation of symptoms and delayed care-seeking remain common in many settings. Addressing these factors through culturally appropriate health education can improve risk awareness, promote timely healthcare utilization, and empower pregnant women with practical strategies to prevent infection and recognize early warning signs [94].

## 7. YF Vaccination in Before and During Pregnancy

Vaccination decisions during pregnancy require careful evaluation of the balance between the risks of wild-type yellow fever infection and the theoretical risks associated with the live-attenuated 17D vaccine. Because protective immunity to YF after infection or vaccination lasts for decades in most people [95], there is a broad window of opportunity for immunological interventions before pregnancy. Vaccination remains the most effective and reliable strategy for preventing YF and reducing disease-related morbidity and mortality in endemic and outbreak settings (Table 4) [1].

Beyond active immunization, passive immunization strategies, including the use of monoclonal antibodies for prophylactic and therapeutic purposes, have emerged as promising complementary tools, particularly for pregnant or immunocompromised individuals in outbreak settings, and may represent a feasible future approach to outbreak mitigation through targeted stockpiling and rapid deployment [96]. Vaccination against YF, including among pregnant women in high-exposure areas, is critical in a condition with a case fatality rate higher than 40% [7]. The currently available live-attenuated vaccine has demonstrated high effectiveness and an adequate safety profile over several decades of use worldwide (Table 5) [2]. It induces robust and long-lasting immunity in most recipients and has been instrumental in controlling transmission and preventing large-scale epidemics [97]. Accumulated evidence indicates that this vaccine is safe for use in the general population and represents a cornerstone of global YF prevention [1,24].

In the context of pregnancy, early theoretical concerns regarding fetal exposure to a replicating virus led to cautious recommendations and classification of the vaccine as one to be used with precaution (Table 5) [98].

However, accumulating observational evidence suggests that YF vaccination during pregnancy is not associated with significant adverse maternal, fetal, or neonatal outcomes [22,102].

Although some studies have reported mild symptoms like headache, fever, and myalgias in up to 19.6% of pregnant women who were inadvertently vaccinated early (at a mean of 5.7 weeks of gestation) in gestation [22] and fetal adverse events, mostly malformations (3.3%), with no differences in several types of major malformations except for Down’s syndrome (3 cases in 304 babies exposed, *p* = 0.003) [80] and spontaneous abortion (<2%) in patients exposed before their last menstrual period or during the first trimester of pregnancy [104,105]. Key quantitative findings from the available observational studies, including cohort size, seroconversion rates, and reported maternal–fetal outcomes, are summarized in the cited references and incorporated into the narrative synthesis presented here. Because the available evidence is primarily observational, vaccination decisions during pregnancy should rely on careful risk–benefit assessment rather than on uniform recommendations.

Multiple studies, including large cohort analyses and post-marketing surveillance data, have failed to demonstrate increased risks of miscarriage, congenital anomalies, stillbirth, preterm birth, or neonatal complications attributable to vaccination [22,52,98,100,101,102]. Importantly, inadvertent vaccination during early pregnancy, including during the first trimester, has not been linked to clinically relevant safety concerns [22,52,98,100,101,102]. Thus, vaccination against YF, including among pregnant women, in high-risk settings, is likely to offer greater benefit than potential risk. It is important to interpret the available evidence on yellow fever in pregnancy with appropriate caution. Much of the available literature consists of observational studies, retrospective analyses, case reports, and post-marketing vaccine surveillance. Prospective controlled studies remain scarce. Consequently, the strength of evidence supporting several conclusions, particularly regarding vaccine safety, immunogenicity, and maternal–fetal outcomes, remains limited. Although accumulated real-world experience and observational data generally support the safety and effectiveness of yellow fever vaccination during pregnancy when clinically indicated, these findings should be interpreted in the context of potential biases inherent to non-randomized data sources, including incomplete follow-up, selection bias, and residual confounding. For this reason, some interpretations presented in this review should be considered hypothesis-generating or based on biological plausibility and indirect evidence rather than definitive causal inference. However, defining precise epidemiological indices to justify including susceptible populations, such as pregnant women, in vaccination campaigns could be challenging.

Regulatory agencies and public health authorities have progressively incorporated this evidence into their guidance [99]. The United States Food and Drug Administration and other regulatory bodies consider the YF vaccine to be safe when administered during pregnancy under appropriate clinical circumstances (https://www.fda.gov/media/76015/download) (accessed on 9 February 2026). Brazil’s National Immunization Program, in a technical document, points out that regarding the use of the YF vaccine in pregnant women, “if it is impossible to postpone vaccination, such as in situations of epidemiological emergency, outbreaks or epidemics, the health service should assess the risk versus benefit of vaccination” (https://www.gov.br/saude/pt-br/vacinacao/calendario) (accessed on 9 February 2026). Consequently, updated clinical guidance and public health messaging could empower primary care and obstetrical providers to incorporate YF vaccination as a key consideration in prenatal care [41,78,106].

**Figure 2:**
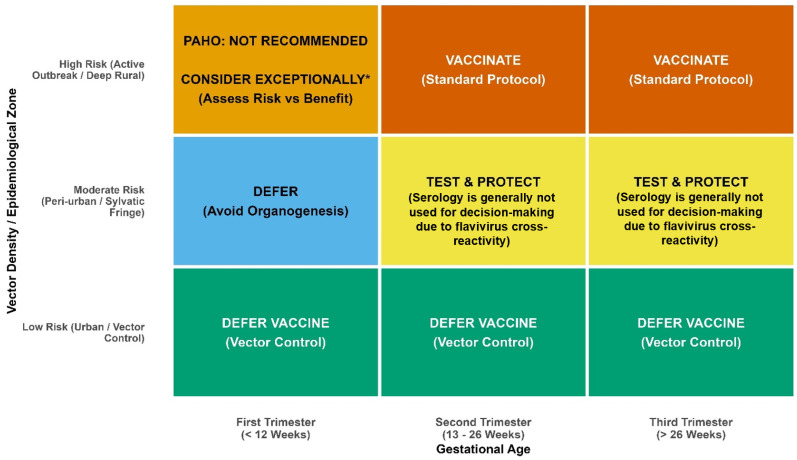
Evidence-informed operational decision matrix for yellow fever vaccination in pregnancy based on observational studies and public health guidance. The matrix integrates gestational age and local transmission intensity to guide vaccination decisions in pregnant women. Recommendations reflect current evidence and regional public health guidance, emphasizing individualized risk–benefit assessment, particularly in high-risk outbreak settings. * <12 weeks: PAHO does not generally recommend vaccination during the first trimester due to theoretical risks. However, in high-risk zones, this protocol proposes its use as an exceptional measure based on a Risk versus Benefit analysis (in which the high probability of maternal mortality outweighs the theoretical risk to the fetus). In all scenarios, vector control and personal protection against vectors are recommended. Testing for immune status assessment is generally not readily available. Even if it were, limitations in serologic interpretation due to cross-reactions among flaviviruses could lead to false-positive results for yellow fever and prevent the vaccine from being recommended.

Accordingly, vaccination decisions should be individualized based on exposure risk and gestational age. Several countries have adopted pragmatic, evidence-based policies that incorporate local epidemiology, gestational age, and individual exposure profiles. Based on these principles and the quantitative findings reported in observational cohorts and vaccination surveillance studies, a risk-based operational framework is proposed to guide vaccination decisions in clinical and outbreak settings (Figure 2) [1]. In areas with active transmission, recurrent outbreaks, or sustained sylvatic circulation, the risk of severe maternal disease, hemorrhagic complications, and maternal mortality clearly outweighs any potential vaccine-related concerns (https://www.minsalud.gov.co/Normatividad_Nuevo/Circular%20Externa%20No%20029%20de%202025.pdf) (accessed on 9 February 2026). In such settings, vaccination represents the most effective means of protecting both maternal and fetal health. Ideally, women of reproductive age in endemic regions who have not been previously immunized against YF should be vaccinated before becoming pregnant. Conversely, in low-risk environments or when exposure can be avoided, deferring vaccination and postponing travel may be considered [1,2,7]. At the population level, strengthening routine pre-pregnancy vaccination in endemic and epidemic-prone areas is a highly effective preventive strategy, analogous to public health approaches used for other infections with major implications for maternal and fetal health, such as hepatitis B and human papillomavirus.

Multiple countries have adopted pragmatic, evidence-based policies reflecting this approach [107]. In the United States, the YF vaccine may be used as a precaution when balancing risks and benefits (https://www.cdc.gov/yellow-fever/hcp/vaccine/index.html) (accessed on 9 February 2026). Pregnant women should avoid or postpone travel to an area where there is a risk of YF. If travel cannot be avoided, discuss vaccination with your healthcare professional. In the United Kingdom, when travel is unavoidable, vaccination in pregnancy should be considered on a case-by-case basis. The possible fetal risks of vaccination should be weighed against the risk to mother and fetus from YF infection, which is associated with significant morbidity and mortality, particularly in immune-naïve individuals (https://uktis.org/monographs/yellow-fever-vaccination-in-pregnancy/) (accessed on 9 February 2026). Similarly, considerations and recommendations are in Canada (https://www.canada.ca/en/public-health/services/publications/healthy-living/canadian-immunization-guide-part-3-vaccination-specific-populations/page-4-immunization-pregnancy-breastfeeding.html) (accessed on 9 February 2026), Australia (https://immunisationhandbook.health.gov.au/contents/vaccine-preventable-diseases/yellow-fever) (accessed on 9 February 2026), France (https://www.pasteur.fr/en/medical-center/vaccines-available-medical-center) (accessed on 9 February 2026), Germany (https://www.auswaertiges-amt.de/resource/blob/2279420/9f78874fa053f8a9cb15c505a5b03ef1/reise-impfempfehlungen-aa-data.pdf) (accessed on 9 February 2026), Spain (https://www.sanidad.gob.es/areas/promocionPrevencion/vacunaciones/programasDeVacunacion/embarazadas/mujeres/docs/Mujeres_edad_fertil_embarazadas_puerperio.pdf) (accessed on 9 February 2026) among other countries.

For example, following national safety assessments, Colombia recommends YF vaccination for pregnant women residing in or traveling to high-risk areas after 12 weeks of gestation (https://www.minsalud.gov.co/Normatividad_Nuevo/Circular%20Externa%20No%20029%20de%202025.pdf) (accessed on 9 February 2026). Ecuador also recommends the use of YF for pregnant women in high-risk areas (https://www.salud.gob.ec/fiebre-amarilla) (accessed on 9 February 2026). Similar risk-based strategies have been implemented in other endemic and non-endemic countries, integrating local epidemiology, individual exposure profiles, and gestational age into vaccination decisions. In Brazil, the vaccination may be recommended for pregnant women when there is a high risk of infection, and vaccination cannot be deferred. This applies to individuals who live in, or will travel to, areas with active transmission, provided that a health service has evaluated them. For travelers, the vaccine should be administered at least 10 days before departure (https://www.gov.br/saude/pt-br/vacinacao/calendario) (accessed on 9 February 2026). When indicated after the first trimester, yellow fever vaccination may be administered alongside inactivated vaccines such as seasonal influenza or COVID-19 vaccines, in accordance with national immunization guidance and individualized clinical assessment [107].

In situations where YF vaccination is recommended, counseling should emphasize the vaccine’s demonstrated safety and effectiveness, the high risk of natural infection, and the substantial benefit of maternal protection, especially during outbreaks. Shared decision-making, supported by clear and transparent information, enhances patient confidence and promotes adherence to preventive measures. Informed consent and appropriate documentation remain essential components of good clinical practice [12,18,49,52,100].

Post-vaccination follow-up should include routine obstetric monitoring and assessment of maternal well-being. Although most vaccinated pregnant women develop an adequate immune response, several studies have shown that seroconversion rates during pregnancy may be lower than in non-pregnant adults and that long-term immunity may be less consistent [41,95,108,109]. In a cohort of 96 infants with available longitudinal follow-up, 51 were born to mothers who were seropositive for YF virus IgG. Among these infants, 36 (70.6%) were IgG-positive at birth, reflecting efficient transplacental transfer of protective maternal antibodies. Longitudinal analyses demonstrated a progressive decline in passively acquired IgG concentrations over time, with substantial interindividual variability in the rate of antibody waning. An additional area of uncertainty is whether high maternal antibody levels in highly endemic settings may transiently blunt infant immune responses to subsequent vaccination or natural exposure, potentially affecting long-term protection. These findings highlight the transient nature of maternally derived immunity and its limited contribution to sustained infant protection [110].

Studies have reported high short-term seroprotection rates following inadvertent YF vaccination during pregnancy, reaching up to 98% in early gestation (2–4 weeks) [22], although substantially lower seroconversion rates have been observed in some cohorts (38.6% at 6 weeks) [41]. Consequently, booster doses after pregnancy are recommended in several countries, including Canada (https://www.canada.ca/en/public-health/services/publications/healthy-living/canadian-immunization-guide-part-4-active-vaccines/page-25-yellow-fever-vaccine.html) (accessed on 9 February 2026), USA (https://www.cdc.gov/yellow-fever/hcp/vaccine/index.html) (accessed on 9 February 2026), Germany [109], Australia, and Colombia (https://www.minsalud.gov.co/Normatividad_Nuevo/Circular%20Externa%20No%20001%20de%202026.pdf) (accessed on 9 February 2026), particularly for women who remain in or return to high-risk areas and were vaccinated during pregnancy [16,68,98,99]. Current recommendations for post-pregnancy booster vaccination are primarily informed by limited immunogenicity data and national vaccination policies rather than by fully established mechanistic evidence.

Breastfeeding considerations should also be addressed. Few case reports have documented transmission of the YF vaccine virus through breast milk during the early neonatal period, leading to severe neurological complications in exposed infants. These observations provide a strong rationale for individualized risk assessment and support recommendations to temporarily interrupt breastfeeding when maternal vaccination is required, particularly in the first weeks of life [111,112]. Although transmission of vaccine-derived virus through breast milk is rare, individualized counseling is appropriate, especially in the early postpartum period. In most cases, the benefits of maternal immunization and continued breastfeeding outweigh potential risks [16,68,98,99]. In women who receive the YF vaccine during breastfeeding, a temporary interruption of breastfeeding for at least 10 days may be considered for infants younger than 6 months, given their increased vulnerability to vaccine-derived viral transmission. In contrast, continued breastfeeding is generally appropriate for infants older than 6 months, in whom the risk of adverse outcomes is substantially lower (Table 4) (https://www.minsalud.gov.co/Normatividad_Nuevo/Circular%20Externa%20No%20029%20de%202025.pdf) (accessed on 9 February 2026).

From a public health perspective, strengthening preconception, adolescent, and routine adult immunization programs is essential to minimize the need for vaccination during pregnancy. Systematic assessment of vaccination history during antenatal care allows early identification of susceptible women and facilitates timely preventive interventions [19,20,101,106].

During mass vaccination campaigns, including those conducted in outbreak settings, pregnant women should not be systematically excluded. Instead, individualized medical assessment, clear operational guidance, and strengthened pharmacovigilance systems are required. The experience from recent outbreaks, including the 2024–2026 epidemic in Colombia, demonstrates that targeted vaccination after the first trimester can be safely implemented when transmission risk is high. It is also important to emphasize that when vaccination is indicated during pregnancy, the standard full-dose YF vaccine should be administered rather than a fractional dose. Dose-sparing strategies using fractional doses have been implemented in outbreak settings. However, fractional dosing has not been adequately evaluated for safety or immunogenicity in pregnant women, and its effectiveness in this population remains uncertain [98]. In other populations, e.g., children, fractional doses do not sustain long-term appropriate antibody titers [113]. Even more, the WHO indicates that until data relevant to specific subgroups become available, children aged < 2 years, individuals known to be HIV-infected, and pregnant women should preferentially be vaccinated using a standard dose [114]. Therefore, full-dose vaccination remains the preferred approach when maternal immunization is required [7].

The recommendations and operational considerations discussed in this article should be interpreted in the context of a narrative synthesis of available evidence rather than as formal evidence-graded guidelines. In several areas, particularly vaccination policies, risk–benefit assessment, and surveillance strategies, the discussion draws on a combination of observational data, existing national and international guidance, and expert-informed interpretation of the literature, given that high-quality prospective evidence remains limited.

In summary, extensive real-world experience and growing scientific evidence support the safety and effectiveness of the YF vaccine during pregnancy. Ultimately, vaccination decisions during pregnancy should be guided by individualized risk–benefit assessment that considers local transmission intensity, maternal exposure risk, and the substantial severity of wild-type yellow fever infection. The vaccine is not contraindicated but should be used with precaution and informed clinical judgment. A risk-based approach that prioritizes maternal protection, supported by appropriate counseling and post-pregnancy booster strategies, represents the most rational and effective framework for preventing YF in pregnant women and their infants in endemic and re-emerging settings [52,115].

Despite the limited and heterogeneous nature of available evidence, this review integrates epidemiological observations, immunological mechanisms, and vaccination data to provide a comprehensive framework for understanding yellow fever in pregnancy and guiding risk-based clinical and public health decision-making.

## 8. Limitations

This perspective has several important limitations relevant to interpreting its findings. First, available evidence on YF in pregnancy remains limited and heterogeneous [16]. Much of the existing literature is derived from outbreak investigations, small case series, retrospective analyses, and isolated case reports. The scarcity of large, prospective, population-based studies limits the ability to accurately estimate incidence, risk factors, and materno-fetal outcomes. Furthermore, because this article was designed as a narrative perspective rather than a systematic review, we did not formally assess the methodological quality or risk of bias of individual studies. As a result, the synthesis presented here reflects a qualitative interpretation of heterogeneous sources of evidence rather than a graded evaluation of evidence strength. In addition, several policy-oriented considerations discussed in this manuscript represent expert-informed interpretations of the available literature and existing public health guidance rather than formally graded recommendations derived from systematic evidence synthesis.

Surveillance systems in many endemic regions do not routinely capture pregnancy status, gestational age, or detailed obstetric and neonatal outcomes. This limitation also prevents reliable estimation of the incidence and outcomes of vertical transmission. We did not attempt to model expected numbers of YF cases or deaths in pregnant women from population pregnancy rates because the available outbreak data lack the pregnancy-specific, age-stratified, and exposure-specific denominators needed for valid estimation. This contributes to underreporting, incomplete case characterization, and potential misclassification. Mild and asymptomatic infections are likely to be overlooked, leading to an underestimation of the true burden of disease. Similarly, current knowledge on vertical and perinatal transmission is based on very few documented cases, limiting generalizability.

Most safety and immunogenicity data on YF vaccination during pregnancy originate from observational studies and post-marketing surveillance. These data sources are inherently subject to selection bias, incomplete follow-up, and residual confounding. In addition, immunogenicity studies are limited in size and geographic scope, which constrains conclusions regarding long-term protection and optimal booster strategies.

An additional limitation reflects the long-standing exclusion of pregnant women from vaccine and clinical research, a practice historically intended to minimize fetal risk but increasingly recognized as ethically problematic and scientifically counterproductive. This paternalistic approach has contributed to persistent evidence gaps, delayed access to effective interventions, and limited data to support informed decision-making. Failure to adequately include pregnant women in research may ultimately undermine autonomy and compromise maternal and fetal health outcomes. Addressing this structural barrier is essential to generate robust, pregnancy-specific evidence for YF prevention and management [116].

An additional limitation relates to global policy frameworks. The WHO Eliminate YF Epidemics (EYE) strategy, covering 2017–2026, does not explicitly address the specific needs of pregnant women [117]. Despite growing recognition of the impact of infectious diseases on maternal and fetal health, pregnancy-specific considerations remain largely absent from major YF control initiatives. As the EYE strategy nears completion, future updates from WHO and PAHO present an important opportunity to integrate targeted protection, surveillance, and research priorities for pregnant women into global YF prevention and response plans [117].

Finally, the recommendations presented in this article are influenced by evolving epidemiological patterns, national policies, and vaccine availability, all of which may change over time. Continued surveillance, standardized reporting, and well-designed prospective studies are essential to address these limitations and strengthen the evidence base for clinical and public health decision-making.

## 9. Conclusions

Despite advances in understanding yellow fever epidemiology and prevention, major gaps remain regarding the clinical course, maternal–fetal outcomes, and optimal vaccination strategies during pregnancy. YF remains a major public health threat in endemic and re-emerging regions, and its impact on pregnant women represents an important yet underrecognized dimension of disease burden. Physiological, immunological, and social changes during pregnancy may influence risk of infection, host responses to infection, and maternal–fetal outcomes, underscoring the importance of targeted prevention, early diagnosis, and integrated clinical management. Despite limited and heterogeneous data, available evidence indicates that YF infection in pregnancy can be associated with significant maternal morbidity, pregnancy loss, preterm birth, and, in rare cases, perinatal transmission.

Vaccination remains the cornerstone of prevention. Accumulated real-world experience and observational studies consistently support the safety and effectiveness of the YF vaccine in pregnant women when used with appropriate clinical judgment. In high-risk settings, the benefits of maternal immunization clearly outweigh theoretical concerns, and risk-based strategies that prioritize maternal protection may be considered. Strengthening preconception immunization, integrating vaccination assessment into antenatal care, and implementing postpartum booster strategies are essential components of comprehensive prevention programs.

Looking ahead, next-generation YF vaccines may further improve the safety profile of immunization in special populations. A Vero cell–derived vaccine based on selected 17D sub-strains with reduced neurotropism is currently under clinical development, and ongoing trials are designed to evaluate whether its immunogenicity is non-inferior to existing yellow fever vaccines (https://clinicaltrials.gov/study/NCT04942210) (accessed on 9 February 2026). This platform may potentially reduce the risk of serious adverse events and could be particularly relevant for pregnant women. However, pregnancy-specific safety data are unlikely to become available in the near future, underscoring the continued need for careful surveillance and dedicated research in this population [103].

From a clinical perspective, improved awareness among healthcare providers is critical to facilitate timely recognition, appropriate supportive care, and multidisciplinary management. Public health systems must enhance surveillance, incorporate pregnancy-specific indicators, and ensure equitable access to preventive and diagnostic services, particularly in vulnerable populations.

Beyond currently available interventions, emerging and innovative approaches for YF prevention and management in pregnancy merit sustained attention and investment, particularly to strengthen preparedness and response in high-risk settings.

Future efforts should focus on establishing prospective pregnancy registries, strengthening pharmacovigilance, and expanding studies of vaccine safety, immunogenicity, and effectiveness in pregnant populations. Despite growing attention to YF in pregnancy, the available evidence remains fragmented, largely drawn from observational reports and small case series. Future research should focus on addressing several critical unanswered questions and priority research areas. First, prospective multicenter cohort studies and pregnancy registries are needed to define better the incidence, clinical spectrum, and maternal–fetal outcomes of yellow fever during pregnancy. Second, mechanistic studies should investigate viral interactions with placental tissues, including potential pathways of vertical and perinatal transmission. Third, larger immunogenicity studies are required to clarify the magnitude and durability of vaccine-induced immune responses during pregnancy and the potential need for postpartum booster strategies. Fourth, improved epidemiological surveillance systems should incorporate pregnancy-specific indicators to quantify disease burden better and inform public health responses. Finally, evaluation of emerging preventive strategies, including next-generation vaccines and passive immunization approaches, will be important to improve protection for pregnant women in endemic and outbreak settings. Such initiatives will help refine clinical guidelines and inform policy decisions. Addressing YF in pregnancy through coordinated clinical, epidemiological, and public health strategies is essential to protect maternal and neonatal health and to strengthen preparedness for future outbreaks in endemic and re-emerging settings.

## Figures and Tables

**Table 1:** Cases, deaths, incidence, fatality, and mortality rates of YF in South America, 2024–2026 (up to 14 March 2026).

Country	Cases	%	Deaths	Population (2026) ^a^	Incidence Rate ^b^	CFR% ^c^	Mortality Rates ^d^
**Colombia ^e^**	175	39.5	77	53,936,000	0.32	44.0	0.143
**Brazil**	128	28.9	52	213,563,000	0.06	40.6	0.024
**Peru**	70	15.8	28	34,218,000	0.20	40.0	0.082
**Venezuela ^e^**	38	8.6	20	28,405,000	0.13	52.6	0.070
**Bolivia**	17	3.8	7	12,413,000	0.14	41.2	0.056
**Ecuador**	11	2.5	8	18,135,000	0.06	72.7	0.044
**Guyana**	4	0.9	1	830,000	0.48	25.0	0.120
**South America**	443	100.0	193	439,700,000	0.10	43.6	0.044

Source: PAHO, https://shiny.paho-phe.org/yellowfever/ (accessed on 14 March 2026). ^a^ Population source: https://data.un.org/ (accessed on 14 March 2026). ^b^ Cases per 100,000 pop. ^c^ Case fatality rate (deaths/cases × 100). ^d^ Deaths per 100,000 pop. ^e^ Three cases imported from Venezuela to Colombia are not reported in any of them [11].

**Table 3:** Key Differential Diagnoses of Yellow Fever in Pregnant Women with Acute Febrile Illness and Hepatic Involvement.

Condition	Key Epidemiologic Clues	Typical Clinical Features	Laboratory Pattern	Pregnancy-Specific Considerations	Initial Diagnostic Tests (Including Tests to Rule out Yellow Fever)	Immediate Management Priorities/Notes
Yellow fever	Travel/residence in endemic or outbreak area; forest/peri-forest exposure; unvaccinated or uncertain status	Acute fever, myalgia, headache; may progress to jaundice, hemorrhage, shock, encephalopathy	AST/ALT elevation (often marked), hyperbilirubinemia, thrombocytopenia, coagulopathy, possible renal dysfunction	Higher risk of hemorrhage and multi-organ failure; fetal compromise secondary to maternal instability	YF RT-PCR during the viremic phase; YF IgM serology after the acute phase (confirm with PRNT when needed); CBC, CMP, PT/INR, creatinine.	Supportive care, ICU if severe; avoid hepatotoxic drugs; multidisciplinary obstetric + critical care
Dengue (including severe dengue)	*Aedes* exposure; urban/peri-urban transmission; recent local dengue activity; household clusters	Fever, retro-orbital pain, rash; warning signs: abdominal pain, bleeding, lethargy; plasma leakage in severe cases	Leukopenia, thrombocytopenia, hemoconcentration; mild–moderate transaminase rise; shock in severe disease	Risk of hemorrhage, preterm birth; fluid management must balance maternal perfusion and pulmonary edema risk	NS1 antigen/RT-PCR (early); IgM/IgG (later); CBC trends and hematocrit; ultrasound for plasma leakage	Careful fluids; avoid NSAIDs; manage bleeding; monitor fetal status in severe illness
Zika virus infection	*Aedes* exposure; sexual transmission possible; outbreak history; travel to endemic areas	Often mild: rash, conjunctivitis, arthralgia, low-grade fever	Usually mild lab abnormalities; occasional leukopenia or mild transaminase elevation	Primary concern is fetal neurodevelopment, especially critical in the first trimester, but it can occur in all trimesters; prolonged maternal viremia can occur	RT-PCR (serum/urine early); serology with cross-reactivity caveats; targeted fetal ultrasound if infected/exposed	Counseling; fetal surveillance per protocol; report and follow pregnancy closely
Chikungunya	*Aedes* exposure, community outbreaks, travel to affected areas	High fever, severe polyarthralgia, rash; may be debilitating	Variable labs; mild transaminase rise possible; thrombocytopenia less prominent than dengue	Maternal infection in the peripartum period is associated with up to 50% with severe neonatal infection; severe maternal pain affects hydration and function	RT-PCR early; serology later; CBC and CMP	Analgesia compatible with pregnancy; hydration; peripartum neonatal readiness if maternal illness near delivery
Oropouche	*Culicoides* (midges) exposure, Travel/residence in endemic or outbreak area,	Similar to dengue The acute phase usually lasts 2 to 7 days, but in cases involving the central nervous system, it can extend up to 2–4 weeks	Lymphopenia, leucopenia, elevated liver enzymes, and thrombocytopenia are common.	Might result in vertical (including intrapartum) transmission, potentially leading to spontaneous abortion and fetal malformation (under study in Latin America) *	RT-PCR during the viremic phase.	Urgent need for research on Oropouche fever’s implications for maternal and neonatal health, alongside strengthened surveillance, vector control, and preventive measures.
Acute viral hepatitis (A, B, C, D, E)	Food/water exposure (A/E); contact with known cases; endemicity; sanitation; outbreaks; blood/sexual exposure (B/C)	Jaundice, malaise, anorexia, nausea; RUQ pain; may be profound fatigue	Marked ALT/AST elevation; hyperbilirubinemia; variable INR; platelets often normal unless severe	Hepatitis E can be severe in pregnancy in some settings; the risk of fulminant hepatic failure	Hepatitis panel (HAV IgM, HBsAg, anti-HBc IgM, HCV Ab/RNA, HDV IgM, HDV Ag, HEV IgM/RNA, where available); PT/INR, CMP	Supportive care; assess for acute liver failure; infection control; specialist input; manage coagulopathy
Malaria (especially *Plasmodium falciparum*)	Travel/residence in a malaria-endemic area; night-time mosquito exposure; lack of prophylaxis	Fever with chills, headache, anemia; severe disease: altered mental status, respiratory distress, hypoglycemia	Anemia, thrombocytopenia; hypoglycemia; acidosis; mild–moderate transaminase elevation; renal dysfunction in severe cases	Higher parasitemia and severe disease risk; fetal loss and low birthweight; treat promptly	Thick/thin smear or rapid diagnostic test; PCR; CBC; glucose; creatinine; lactate if severe, liver enzymes	Start pregnancy-appropriate antimalarial urgently; manage hypoglycemia and anemia; fetal monitoring in severe cases
Leptospirosis	Exposure to floodwater/rodents/animal urine; occupational risk; outbreaks after heavy rains	Fever, severe myalgias (calves), conjunctival suffusion; jaundice in severe cases (Weil disease)	Elevated bilirubin, creatinine; thrombocytopenia; mild–moderate transaminase elevation, leukocytosis, neutrophilia	Can mimic viral hepatitis/YF; may cause fetal loss; antibiotics reduce severity	*Leptospira* PCR (early) or serology; CMP, CBC, urinalysis; consider CXR if pulmonary symptoms	Start appropriate antibiotics promptly; supportive care; assess renal/pulmonary involvement
Acute cholangitis/biliary disease	History of gallstones; biliary colic; prior episodes; pregnancy-related cholestasis risk	RUQ pain, fever, jaundice; nausea/vomiting; may have hypotension if severe	Cholestatic pattern (ALP, GGT, bilirubin); variable AST/ALT; leukocytosis	Pregnancy increases gallstone disease; imaging choice matters	RUQ ultrasound; CBC; CMP; ALP, GTP, AST, ALT, Bilirrubins; blood cultures if septic	Early antibiotics and source control; surgical/ERCP consult; maternal stabilization
HELLP syndrome/severe preeclampsia with hepatic involvement	Hypertension/proteinuria or symptoms after 20 weeks; prior preeclampsia; multiple gestation	RUQ/epigastric pain, headache, visual symptoms; nausea/vomiting; may have edema	Hemolysis, elevated liver enzymes, low platelets; proteinuria; possible AKI	Obstetric emergency; can resemble viral hepatitis/YF; timing and BP clues are key	BP measurement; urine protein; CBC with smear; AST/ALT; LDH; creatinine; coagulation profile	Stabilize mother; magnesium sulfate as indicated; antihypertensives; plan delivery based on severity/GA
Acute fatty liver of pregnancy (AFLP)	Typically, the 3rd trimester, risk with multiple gestation, prior AFLP, may overlap with preeclampsia	Nausea/vomiting, abdominal pain, jaundice; encephalopathy possible	Moderate transaminase elevation, hypoglycemia, elevated ammonia, coagulopathy, renal dysfunction	Rapidly progressive; maternal–fetal mortality without urgent care	Glucose; CMP; PT/INR; ammonia if available; CBC; consider Swansea criteria	ICU-level supportive care; correct hypoglycemia/coagulopathy; expedite delivery once stabilized
Herpes simplex virus hepatitis	Primary HSV infection; mucocutaneous lesions may be absent; third trimester	Fever, abdominal pain, encephalopathy; often anicteric	Marked AST/ALT elevation, thrombocytopenia, leukopenia, and coagulopathy	High mortality if untreated; often misdiagnosed	HSV PCR (blood/lesions); liver enzymes; CBC	Immediate empiric acyclovir; ICU support
Sepsis from other causes (e.g., pyelonephritis, pneumonia)	Urinary symptoms; respiratory symptoms; risk factors for infection; recent procedures	Fever, tachycardia, hypotension; organ dysfunction; may have focal symptoms	Leukocytosis or leukopenia; lactate elevation; LFTs may rise in shock liver	Pregnancy alters vitals and labs; fetal distress can be an early sign of maternal sepsis	Blood/urine cultures; lactate; CBC; CMP; imaging guided by symptoms	Early antibiotics, fluids, source control; fetal monitoring in viable pregnancy; ICU if needed

This table summarizes key conditions considered in the differential diagnosis of suspected yellow fever in endemic settings and highlights initial laboratory tests that guide early diagnostic evaluation before confirmatory testing is available. * Up to 19 March 2026, no studies have proved the causal relationship between Oropouche maternal infection and birth defects; it remains under study [9,62,63,64]. In severe cases, reactive hemophagocytic lymphohistiocytosis has also been reported as a complication of YF, reflecting profound immune dysregulation and systemic inflammation. This condition should be suspected in patients with persistent fever, cytopenias, hyperferritinemia, and progressive organ dysfunction. AFLP, acute fatty liver of pregnancy; ALT, alanine aminotransferase; AST, aspartate aminotransferase; BP, blood pressure; CBC, complete blood count; CMP, comprehensive metabolic panel; CXR, chest X-ray; ERCP, endoscopic retrograde cholangiopancreatography; GA, gestational age; GGT, gamma-glutamyl transferase; HAV, hepatitis A virus; HBsAg, hepatitis B surface antigen; HBc, hepatitis B core; HCV, hepatitis C virus; HDV, hepatitis D virus; HEV, hepatitis E virus; HSV, herpes simplex virus; ICU, intensive care unit; IgG, immunoglobulin G; IgM, immunoglobulin M; INR, international normalized ratio; LDH, lactate dehydrogenase; LFTs, liver function tests; NS1, non-structural protein 1; PCR, polymerase chain reaction; PRNT, plaque-reduction neutralization test; PT, prothrombin time; RNA, ribonucleic acid; RT-PCR, reverse transcription polymerase chain reaction; RUQ, right upper quadrant; YF, yellow fever.

**Table 4:** Risk–Benefit Framework for Yellow Fever Vaccination in Pregnant Women.

Scenario	Transmission Risk at Destination/Residence (Based on WHO/CDC)	Maternal Exposure Profile	Gestational Age	Vaccination Recommendation	Rationale	Suggested Follow-Up/Additional Measures
Active outbreak in the area of residence	High	Resident in outbreak-affected municipality; routine daily exposure	Any trimester	Vaccinate after risk–benefit counseling (ideally >12 weeks of gestation)	Risk of severe yellow fever and maternal mortality likely outweighs theoretical vaccine risk	Enhanced mosquito avoidance; close maternal–fetal monitoring; report to pregnancy registry if available
Active outbreak; unavoidable travel (work/humanitarian/essential)	High	Unavoidable travel to outbreak zone; prolonged stay or rural/peri-forest exposure	Any trimester	Vaccinate (preferably before travel) (ideally >12 weeks of gestation)	Substantial exposure risk; postponement not feasible	Pre-travel counseling; mosquito precautions; plan for access to care; consider post-vaccination serology if policy supports
Endemic area without current outbreak; sustained sylvatic circulation	Moderate	Resident or frequent travel to forest fringe/riverine/rural areas	2nd–3rd trimester	Consider vaccination if exposure is ongoing and significant	Ongoing exposure increases cumulative risk; later gestation may simplify obstetric monitoring	Strengthen vector protection; routine antenatal follow-up; document shared decision-making
Endemic area without current outbreak; low exposure	Low–Moderate	Urban residence, limited outdoor exposure, good vector control	Any trimester	Defer vaccination; emphasize vector precautions	Lower short-term exposure risk; avoid live vaccine when not clearly needed	Reassess if epidemiology changes; reinforce repellents, screens, bed nets
Non-endemic area; traveler considering discretionary travel to an endemic region	Moderate	Elective tourism or flexible itinerary	2nd–3rd trimester	Avoid travel or postpone; if travel cannot be postponed, individualize vaccination decision	Best prevention is avoiding exposure; the vaccine is live-attenuated	If travel proceeds: strict mosquito precautions; select lower-risk routes/locations; ensure travel insurance and care access
First trimester in a moderate-risk setting	Moderate	Planned travel or intermittent exposure; ability to postpone or modify exposure	1st trimester	Prefer deferral and exposure avoidance when feasible	Theoretical concerns are greatest early in gestation; many exposures can be mitigated by postponement	Reassess in the 2nd trimester if risk persists; prioritize non-pharmacologic protection
Late pregnancy with high exposure and limited access to care	High	Rural residence; limited healthcare access; intense vector exposure	3rd trimester	Vaccinate; prioritize maternal protection	Severe maternal disease near term can be catastrophic; protecting the mother is central to fetal survival	Plan delivery site with higher-level care; continuous fetal surveillance if clinically indicated
Inadvertent vaccination before pregnancy is recognized	Unrelated to risk scenarios	Vaccinated peri-conception or early pregnancy (unintentional)	Any	No intervention required solely for vaccination; provide reassurance	Most evidence does not show consistent severe adverse outcomes from inadvertent vaccination	Routine obstetric care; offer targeted ultrasound per local practice; report to pharmacovigilance
Previously vaccinated (documented or credible history)	Depends	Prior YF vaccination before conception; no contraindications	Any	No revaccination in pregnancy in most cases	Prior vaccination likely provides durable protection; avoid unnecessary live vaccine exposure	Verify documentation if possible; counsel on mosquito protection; consider booster only if policy requires and risk is extreme
Uncertain vaccination history; high-risk exposure	High	No records; high-risk residence/travel; limited time for verification	Any trimester	Treat as unvaccinated and consider vaccination if exposure is substantial (ideally >12 weeks of gestation)	Delay may increase infection risk; the decision must be pragmatic in emergencies	Document uncertainty; consider serology if available and timely; reinforce mosquito precautions
Postpartum—breastfeeding mother needs vaccination due to outbreak/travel	High	Breastfeeding with imminent exposure risk	Postpartum	Vaccinate; counsel on the rare risk of vaccine virus transmission via breast milk. Temporary interruption of breastfeeding for at least 10 days should be recommended for infants younger than 6 months.	Maternal protection is important; breastfeeding transmission appears rare but possible	Shared decision; consider temporary interruption of breastfeeding for a defined period if local guidance recommends; monitor the infant
Mass vaccination campaign during the outbreak	High	Pregnant woman in campaign area; varying exposure levels	Any trimester	Individual risk assessment within campaign framework; vaccinate when exposure is high (ideally >12 weeks of gestation)	Population-level control vs. individual considerations; avoid blanket exclusion if outbreak risk is significant	Clear consent processes; pregnancy registry; strengthened adverse event surveillance; tailored counseling

**Table 5:** Safety of Yellow Fever Vaccine in Pregnancy by Trimester [52,98,99,100,101,102,103,104].

Trimester of Pregnancy	Safety Considerations	Available Evidence	General Recommendation
**First trimester ** **(0–12 weeks)**	Increased caution. Possible theoretical risk of teratogenicity and miscarriage (as it is a live attenuated virus vaccine). Animal studies have shown placental transmission, although evidence in humans is limited.	Retrospective studies have shown no significant increase in congenital malformations; however, the number of cases is limited.	Avoid if possible. Only manage if the risk of exposure is high and unavoidable. Perform detailed prenatal follow-up.
**Second trimester ** **(13–26 weeks)**	Lower theoretical risk compared to the first trimester. A similar rate of adverse events has been reported in the vaccinated general population.	Most studies with positive data include women vaccinated in this trimester. There has been no evidence of an increase in miscarriage or fetal adverse effects.	It may be considered if the risk of exposure is high. Accompanied by adequate obstetric monitoring.
**Third trimester ** ** (27 weeks–delivery)**	Low risk of teratogenic effects, but considered a risk of vertical transmission with the appearance of antibodies in the newborn.	IgM antibodies have been documented in neonates of vaccinated mothers, with no evidence of clinical disease.	It may be given if there is a clear indication. Low risk, but it is recommended to observe the newborn after birth.

## Data Availability

No new data were created or analyzed in this study. Data sharing is not applicable to this article.
